# Mutagenicity and Repair of Acrolein Adduct to Cytosine

**DOI:** 10.3390/ijms27010071

**Published:** 2025-12-21

**Authors:** Małgorzata Dylewska, Sławomir Kasperowicz, Beata Sokołowska, Agnieszka M. Maciejewska

**Affiliations:** 1 Institute of Biochemistry and Biophysics, Polish Academy of Sciences, Pawińskiego 5a, 02-106 Warsaw, Poland; mdylewska@ibb.waw.pl (M.D.); slakas@ibb.waw.pl (S.K.); 2 Mossakowski Medical Research Institute, Polish Academy of Sciences, Pawińskiego 5, 02-106 Warsaw, Poland; beta.sokolowska@imdik.pan.pl

**Keywords:** DNA repair, acrolein adduct, mutagenicity, Ada response, AlkA glycosylase, AlkB dioxygenase, molecular modeling

## Abstract

Acrolein, a ubiquitous environmental pollutant, is also formed endogenously as a metabolite under oxidative stress conditions. Its adduct to cytosine, 3,N^4^-α-hydroxypropanocytosine (HPC), has recently been shown to be an in vitro substrate for the AlkB dioxygenase. Using a set of indicator plasmids modified with acrolein, we provide evidence that HPC is a mutagenic non-instructional lesion that predominantly induces C→A transversion, and to a lesser extent C→T and C→G base substitutions. HPC is efficiently repaired in vivo by AlkB, even without induction of the adaptive response. However, the mutation frequency did not differ between the wild-type and AlkA-deficient strains, and AlkA glycosylase fails to excise in vitro the acrolein-modified cytosine from the T_22_(HPC)_3_ oligodeoxynucleotide, both indicating that HPC is not a substrate for AlkA. Based on molecular modeling, we further examined the potential differences in the hydrolytic suspensibility of a known AlkA substrate, the acrolein adduct to adenine (HPA), and the cytosine adduct (HPC) at the glycosylase active site. Analysis of both structural and electrochemical properties indicates that, despite an identical type of modification within an equivalent chemical context, including comparable geometry and topology, the glycosidic bond in HPC is considerably less susceptible to hydrolysis than that in HPA.

## 1. Introduction

Acrolein (propenal, CH_2_CHCHO) is a volatile, highly reactive, and toxic α,β-aldehyde. Biologically, it is one of the most harmful environmental pollutants due to its strong electrophilic nature, which allows it to bind to cellular macromolecules such as proteins, lipids, and DNA [1]. In addition to environmental exposure, acrolein is produced endogenously in the human body as a byproduct of lipid peroxidation and the metabolism of amino acids (e.g., threonine), and polyamines [2]. This internal production contributes to oxidative stress and inflammation, particularly in conditions of oxidative damage or disease [3]. Exposure to acrolein through cigarette smoke, car exhaust, industrial emissions, and even food can cause severe side effects [4,5,6]. Chronic exposure has been linked to cardiovascular diseases, asthma, chronic obstructive pulmonary disease, and neurodegenerative disorders [7].

**Figure 1:**
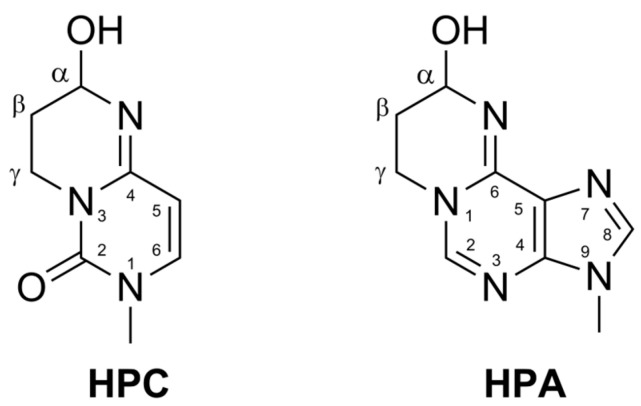
Structures of acrolein adducts to cytosine, 3,N^4^-α-hydroxypropanocytosine (HPC), and adenine, 1,N^6^-α-hydroxypropanoadenine (HPA). Note that the Cα atom of the hydroxypropano ring is asymmetric.

Acrolein easily forms exocyclic adducts, known to distort the DNA helix, interfere with replication and transcription, and, if not efficiently repaired, lead to mutagenesis [8]. Furthermore, acrolein-DNA adducts can inhibit DNA repair enzymes, including those involved in base excision repair and nucleotide excision repair, amplifying the mutagenic and cytotoxic effects [9]. Acrolein primarily forms adducts at guanine residues, namely 1,N^2^-α-hydroxypropanoguanine and 1,N^2^-γ-hydroxypropanoguanine (HPGs) [10], which are specifically and quantitatively repaired by *E. coli* nucleotide excision repair (NER) enzymes UvrA, UvrB, and UvrC, functioning as the UvrABC nuclease [5,11,12]. In addition to the guanine adducts, minor cyclic acrolein adducts 1,N^6^-α-hydroxypropanoadenine (HPA) and 3,N^4^-α-hydroxypropanocytosine (HPC, see Figure 1 for structures) were also identified in DNA [13,14,15,16,17,18] and proven to be repaired by AlkB [19,20,21].

All living organisms possess a network of DNA repair mechanisms that safeguard genomic stability. Among these, the adaptive (Ada) response represents a well-characterized inducible system. This phenomenon describes the capacity of microorganisms to acquire resistance and adapt to elevated concentrations of a mutagen following prior exposure to sublethal levels. In *E. coli*, the Ada operon comprises four genes *(ada*, *alkA*, *alkB*, and *aidB*) whose expression is induced by non-toxic doses of direct-acting methylating agents (reviewed in [22,23]).

The AlkA protein (3-methyladenine DNA glycosylase II; EC 3.2.2.21) is a monofunctional DNA N-glycosylase that initiates the base excision repair (BER) pathway by removing alkylated bases from DNA. AlkA displays broad substrate specificity, catalyzing the excision of a variety of alkylation products, including N^3^- and N^7^-alkyl purines, O^2^-alkyl pyrimidines—its primary substrate being 3-methyladenine—as well as deaminated bases such as hypoxanthine and xanthosine, and oxidative lesions such as oxanine and 5-formyluracil [24,25]. Our in vivo analyses have excluded AlkA involvement in the repair of 1,N^6^-ethenoadenine (εA), 3,N^4^-ethenocytosine (εC), and its precursor 3,N^4^-α-hydroxyethanocytosine (HEC) [26,27]. We have recently described another AlkA substrate, 1,N^6^-α-hydroxypropanoadenine, the acrolein adduct to adenine [21]. Mechanistically, AlkA acts by flipping the damaged base out of the DNA helix into its active site. Within the catalytic pocket, a conserved aspartate residue activates a water molecule, which then performs a nucleophilic attack on the N-glycosidic bond connecting the alkylated base to the deoxyribose sugar. This reaction cleaves the bond, releasing the damaged base and leaving behind an abasic (AP) site in the DNA [25,28,29,30]. AlkA preferentially targets electron-deficient purines with weakened glycosidic bonds, which are readily cleaved under acidic conditions [31]. AlkA’s enzymatic selectivity may be attributed to the chemical lability of the glycosidic bond in these destabilized substrates rather than to a selective recognition and binding of modified bases by the enzyme. It was proposed that the glycosylase binds both substrate and non-substrate bases, but only nucleotides with weakened glycosidic bonds are cleaved efficiently [32]. Supporting this hypothesis, alkylated bases such as 3,N^4^-ethenocytosine and 1,N^6^-ethenoadenine, which are not electron-deficient and possess stable glycosidic bonds, are not easily excised from DNA by AlkA [33,34,35], a finding we confirmed in vivo [26,27]. The *E. coli* AlkB enzyme (EC 1.14.11.33), a member of the α-ketoglutarate- and iron(II)-dependent dioxygenase superfamily, is evolutionarily conserved across nearly all domains of life, including many viruses [36,37,38]. AlkB and its homologs catalyze the oxidative demethylation of alkylated bases, thereby reversing methylation damage at the N1 position of purines and the N3 position of pyrimidines, thereby restoring the native nucleobases [39,40]. The best AlkB substrates, m^3^C and m^1^A, exist in a cationic form at physiological pH, while those neutral at pH 7 (e.g., m^3^T and m^1^G) are repaired very poorly [41,42,43]. As previously shown, efficient repair of exocyclic adducts such as εC, εA, HEC, HPC, and HPA occurs at pH levels that promote their protonated forms [19,20]. It was also demonstrated that ACR adducts to guanine, α-hydroxypropano-dG (α-OH-PdG) and γ-hydroxypropano-dG, which are neutral under physiological conditions, are processed by AlkB. Their repair reactions are complex, involving multiple intermediates and overlapping pathways that converge at several points. Notably, only α-OH-PdG was partially dealkylated, recovering dG [44]. Interestingly, QM/MD calculations (combining molecular dynamics with quantum mechanics) provided unique mechanistic insights into AlkB’s catalytic reaction pathways with ss-DNA containing complex guanine adducts [45].

Our in vitro and in silico analyses employing substrates with different pK_a_ values substantiated the hypothesis [42,46] that AlkB preferentially recognizes and repairs substrates in their protonated (cationic) state [19,47]. We have also confirmed in vivo AlkB participation in the repair of εC and εA lesions [26,27], although not in the removal of their respective triphosphate derivatives from the nucleotide precursor pool [48]. Furthermore, for the first time, we demonstrated that AlkB efficiently removes the εC precursor–HEC [26], as well as both the minor acrolein-derived adducts 3,N^4^-α-hydroxypropanocytosine [19] and 1,N^6^-α-hydroxypropanoadenine (HPA) [20,21]. All the adducts are repaired completely. Whereas HPA was well characterized in vitro, in vivo, and in silico as a good substrate of both Ada response-induced repair enzymes, AlkA glycosylase and AlkB dioxygenase, and HPC as an efficiently repaired substrate of AlkB in vitro and in silico, little data available suggests that AlkA does not excise HPC [49].

In this study, we present data on the mutagenic potential and repair mechanisms of acrolein adducts to cytosine. By using wild-type *E. coli* alongside mutants deficient in AlkB and/or AlkA, we assessed the roles of these enzymes in the repair process. The results indicated that AlkB plays a significantly more important role in HPC repair than AlkA. Additionally, our findings show that the purified AlkA protein does not excise acrolein-modified cytosine from an oligodeoxynucleotide. Through molecular modeling, we clarify potential differences in hydrolysis behavior between HPA and HPC at the AlkA active site. Notably, despite certain similarities, the glycosidic bond in HPC is considerably less susceptible to hydrolysis than that in HPA.

## 2. Results and Discussion

### 2.1. In Vivo Repair of HPC by E. coli AlkA Glycosylase and AlkB Dioxygenase

To study mutagenicity and repair of HPC in vivo, we used the unique test system previously employed for studies of acrolein and chloroacetaldehyde (CAA) mutagenicity [20,21,26,27]. The system consists of a set of pIF plasmids [50] carrying lactose operon alleles from strains CC101–106. These constructs enable monitoring of *Lac^+^* revertants that arise from single-base substitutions (reverse mutations) at codon 461, thereby restoring the wild-type β-galactosidase gene [51]. The three plasmids used, pIF102, pIF103, and pIF104, were indicative of the GC→AT, GC→CG, and GC→TA substitutions. The plasmids were treated with various concentrations of ACR in vitro under conditions that prevent the modification of thymine residues [52]. They were then purified and introduced into *E. coli* wt, and *alkA*, *alkB*, and double *alkAalkB* mutants via electrotransformation. While all plasmid bases but thymine may have undergone modification, the only cytosine-guanine pair at codon 461 was monitored. This method enabled the simulation of endogenously produced HPC while avoiding the detrimental effects of ACR on cells. To study HPC repair, we employed conditions of induced and uninduced Ada response.

The results from studies using pIF102, pIF103, and pIF104 concern the mutagenicity and repair of a mixture of acrolein adducts to G-C pairs. The modified nucleotides HPC and HPGs arise from alterations to cytosine and guanine, respectively. Although they differ in extent, both HPGs are mutagenic. However, HPGs are repaired via the nucleotide excision repair (NER) pathway, with no evidence for involvement of the base excision repair pathway [12]. Repair of HPGs by AlkB dioxygenase is ineffective [20,44], and our preliminary in silico analyses suggest that AlkA does not process them because of the increased stability of their glycosidic bonds. Therefore, the potential role of HPGs in the observed mutagenic effects, although not absolutely excluded, is negligible.

#### 2.1.1. The Transformation Efficiency

Depending on the batch of competent cells, the transformation efficiency of bacteria with unmodified, control plasmids ranged from 1× to 2 × 10^6^ cfu/µg DNA. This efficiency decreased with increasing ACR concentration used for plasmid modification, dropping to 2 × 10^4^ at 50 mM and 25 mM ACR for the single and double mutants, respectively. We previously observed a similar effect for the mutagenesis induced by chloroacetaldehyde and acrolein [20,21,26,27]. We believe that, in both cases (i.e., ACR and CAA), this phenomenon reflects the extent of modification-induced damage to plasmid DNA and the transformed cells’ inability to repair it, as discussed previously [26].

#### 2.1.2. The Mutation Frequency

**Figure 2:**
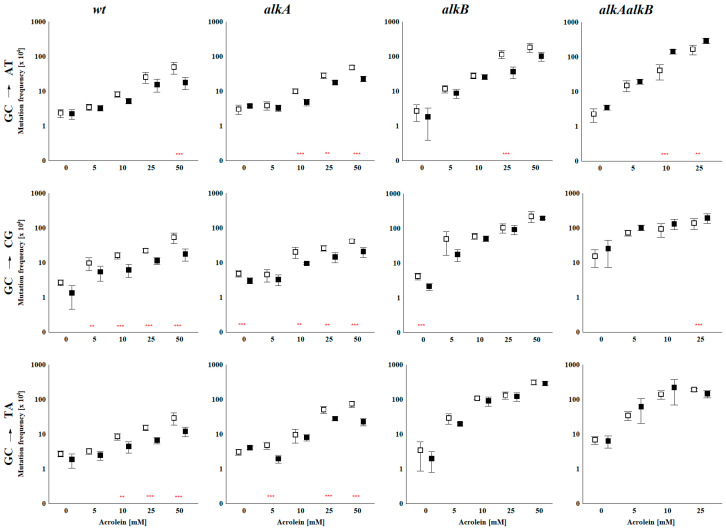
Frequencies of HPC-induced *Lac^+^* reversions in the plasmid’s codon 461 of the *lacZ* gene. Plasmids pIF102, which indicate C→T transitions; pIF103, which indicate C→G transversions; and pIF104, which indicate C→A transversions, were treated with increasing doses of ACR in vitro and then replicated in wild-type, *alkA*, *alkB*, and *alkAalkB E. coli* strains. On the *x*-axis, the concentration (mM) of ACR used for plasmid modification is shown; on the *y*-axis, mutation frequency (MF), expressed as the number of revertants per 10^4^ transformed cells, is presented. The vertical panels display MF results for different substitutions within a specific strain, while the horizontal panels compare MF results for a particular substitution across the studied strains. Mean ± standard deviation was calculated from at least three independent experiments. White and gray bars represent MF values in bacteria with uninduced and induced Ada responses, respectively. The statistical significance of differences between these values: ** *p* < 0.01, and *** *p* < 0.001.

The mutation frequencies for all three substitutions are shown in Figure 2. Numerical data and additional statistics regarding the significance of differences between various strains compared pairwise between variants with adapted or non-adapted (induced or uninduced) Ada response, respectively, are presented in Supplementary Tables S1–S3.

##### Spontaneous Mutation Frequency

The effects of the *alkA*, *alkB*, and *alkAalkB* mutations on the spontaneous mutation frequency varied slightly among the different plasmids tested. The highest effect was observed for the pIF103 plasmid, where the SMF was about twice as high in single mutants and up to six times higher in the double *alkAalkB* mutant compared to wild-type strains in non-adapted cells. In adapted cells, the SMF was two to three times higher for single mutants and twenty times higher for the double mutant. However, it is important to note that the standard deviations (SDs) for the double mutant were relatively high.

##### HPC-Induced Mutations

The frequency of mutations (MF) caused by HPC presence in the plasmid increased with increasing ACR doses used for in vitro modification across all three plasmids and all strains tested, indicating that the adduct is mutagenic. Importantly, the effect of MF increase is much more pronounced in the *alkB* mutant than in the wild-type and AlkA-deficient strain. Closer inspection reveals that in the case of pIF102 and pIF103 plasmids, there is no visible, statistically significant difference in MF between the wt and *alkA* strain, adapted and non-adapted alike, suggesting that AlkA glycosylase is not involved in HPC repair. In pIF104, MFs for the *alkA* strain are 2–3 times higher than for the wild-type. The reason for this phenomenon is unclear. However, the effect is visible only at higher acrolein concentrations used for plasmid modifications (25 and 50 mM), being lower than the difference between the AlkB-deficient strain and the wild-type.

In contrast to *alkA*, the MF for the AlkB-deficient strain is consistently 4–5 times higher for plasmids pIF102 and pIF103, even at low ACR concentrations, and 10 times higher for pIF104 than for the wild-type in the case of the non-adapted strain (Figure 2).

The data indicate that AlkB repairs HPC even at its low constitutive level. The differences become even more pronounced in the adapted strains, reaching up to 20-fold higher for pIF104. These data strongly support the involvement of AlkB dioxygenase in HPC repair. The results provide strong evidence that the AlkB dioxygenase participates in HPC repair, thereby confirming our earlier in vitro finding that HPC is efficiently repaired by AlkB [19]. Importantly, the effect of induction of the Ada response on MF is visible for all plasmids, but only for wild-type and *alkA* strains, in both of which the AlkB dioxygenase activity is increased. No such effect is observed for the AlkB-deficient strain, in which AlkA glycosylase level is increased. It again convincingly demonstrates that AlkB, but not AlkA protein, is involved in HPC lesion repair. Double *alkAalkB* mutant strain, devoid of activity of both enzymes, shows a mutation frequency level close to that of the *alkB* strain, although usually higher. The induction of the Ada response not only did not decrease its MF, but actually increased it. MMS pretreatment made the strain more susceptible, resulting in a higher MF than in the non-adapted double mutant. A similar effect was previously noted for CAA and ACR mutagenicity [20,21,26,27], which we attributed to the activation of the SOS and UVM responses (see [26] for details). We posit that the same explanation applies here.

As in previous studies, modifications induced by 50 mM ACR were markedly higher in the double *alkAalkB* mutant, resulting in a very low transformation efficiency. As a result, virtually no mutants grew on minimal lactose plates, precluding the determination of the mutation frequency.

Although the differences in relative mutation frequencies observed in the *alkB* strain are not substantial, HPC induces predominantly C→A transversions, followed by C→T transitions and C→G transversions. Accordingly, it should be classified as a non-instructive lesion. C→A transversions arise through the incorporation of thymine opposite HPC in the DNA, a pairing that appears to cause minimal distortion of the canonical DNA structure. The mechanism by which HPC generates these mutations requires further investigation using advanced experimental approaches, including detailed analyses of polymerase-DNA-lesion interactions supported by computational molecular modeling.

The results obtained for all three plasmids demonstrated that the products of the *alkB*, but not the *alkA* gene, are engaged in repairing the ACR adduct to cytosine. Moreover, AlkB repairs HPC in vivo, even without prior induction of Ada. The same effect was observed for HPA and some ethenoadducts, and, in the case of HPA, also for AlkA glycosylase [20,21,26,27,53]. Thus, beyond enabling the cell to manage high levels of exogenous alkylating agents, the Ada-response enzymes may also play a constitutive role in controlling endogenous DNA adducts.

**Figure 3:**
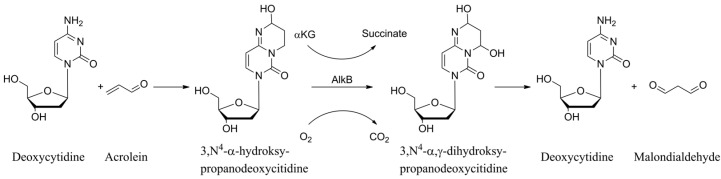
Schematic representation of the reaction leading to in vivo and in vitro formation of HPC and its AlkB-mediated complete repair.

Drawing on the acrolein–cytosine reaction described in references [13,14], the established catalytic mechanism of AlkB outlined in [39,40,53,54], and the observation that malondialdehyde is the final product released during AlkB-mediated repair of ACR-modified guanine [44], as well as the fact that HPC is repaired completely [19], Figure 3 illustrates the formation of HPC in DNA and its subsequent repair by AlkB.

### 2.2. HPC Is Not Excised by AlkA Glycosylase In Vitro

**Figure 4:**
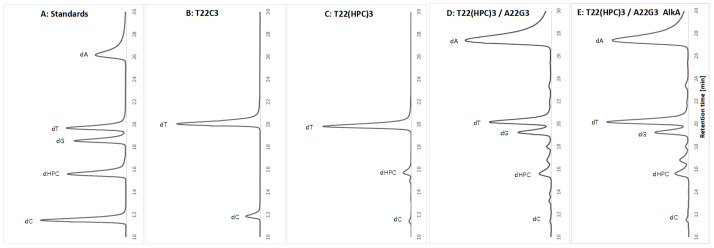
In vitro excision of HPC by AlkA glycosylase. (**A**) Standards for the expected deoxynucleosides with their retention times (RT): dC at 11.5 min, dHPC at 15.7, dG at 18.6, dT at 19.7, and dA at 26.3 min, respectively. (**B**–**E**) Results of enzymatic digestion for the studied oligodeoxynucleotides: (**B**) Unmodified single-stranded T_22_C_3_; (**C**) Single-stranded T_22_(HPC)_3_, which is T_22_C_3_ treated with ACR; (**D**) Double-stranded T_22_(HPC)_3_/A_22_G_3_, created by annealing T_22_(HPC)_3_ to its complementary strand A_22_G_3_; and (**E**) Double-stranded T_22_(HPC)_3_/A_22_G_3_ after the reaction with AlkA. Each deoxynucleoside is labeled above the corresponding peak. Throughout the study, chromatograms (**B**–**E**) were generated, and the retention times of individual peaks fluctuated slightly due to variations in separation conditions. The minor, unlabeled side products in the reaction exhibit UV spectra similar to those of the expected deoxynucleosides; however, we have not determined their identities.

In our recent study [21], we demonstrated that AlkA glycosylase removes ACR adenine adducts (HPA) from DNA. However, using the same in vitro assay, we were unable to confirm AlkA’s ability to excise HPC as shown in Figure 4. The HPC adduct was generated under controlled conditions, allowing selective modification of cytosine residues only [52] in a single-stranded oligodeoxynucleotide that contained three cytosine (C) and 22 thymine (T) residues. As expected, the unmodified oligodeoxynucleotide contained only the dT and dC nucleosides (Figure 4B). Following ACR treatment, a third peak corresponding to dHPC appeared (Figure 4C). The extent of cytosine modification was estimated by comparing the HPLC profiles of nucleotides derived from the unmodified oligodeoxynucleotide to those from the oligodeoxynucleotide following ACR treatment. Since the molar absorption coefficient of HPC is unknown, these calculations are approximate. Considering that the absorption maxima of C and HPC differ (269 nm for C and 281 nm for HPC), we concluded that approximately 70% of cytosines were converted to HPC. The partially modified T_22_(HPC)_3_ oligodeoxynucleotide was then annealed with a complementary unmodified strand to form a double-stranded substrate for AlkA. In the control reaction, which did not include the enzyme, and after treatment with AlkA, the dHPC peak remained unchanged (Figure 4D vs. Figure 4E).

Based on the mutagenicity test results, we decided not to optimize the reaction. However, we confirmed the activity of AlkA glycosylase on methylated substrates [21]. Furthermore, under the same conditions (and, in fact, performed in parallel), the AlkA reaction with a substrate containing HPA confirmed that HPA is indeed a substrate for AlkA [21]. Interestingly, one of the pioneering studies on AlkA glycosylase involvement in the excision of acrolein adducts (originating from our laboratory) demonstrated that AlkA excises some undefined hydroxypropano-adducts, since single-strand breaks were formed in plasmid DNA pretreated with ACR upon combined action of AlkA and an endonuclease [55]. Later, it was found [49] that the excision of HPC by AlkA is highly inefficient, approximately two orders of magnitude lower than that by the *E. coli* glycosylase Mug [34]. In light of our current discoveries, they likely observed HPA excision, although HPGs cannot be ruled out.

### 2.3. Molecular Modeling of HPC Poses in the Substrate-Binding Pocket of AlkA Glycosylase

The recently characterized acrolein adduct to adenine, 1,N^6^-α-hydroxypropanoadenine, is efficiently repaired by both AlkA glycosylase and AlkB dioxygenase [20,21]. In contrast, the acrolein adduct to cytosine examined in this study, 3,N^4^-α-hydroxypropanocytosine, despite its structural similarity to HPA, serves as a substrate exclusively for AlkB. Considering that AlkA’s substrate selectivity is primarily determined by the chemical lability of the glycosidic bond [25,28,29,30,31,32], we conducted molecular modeling to compare how the two acrolein adducts are accommodated within the AlkA substrate-binding pocket to exclude other sources of AlkA selectivity.

**Figure 5:**
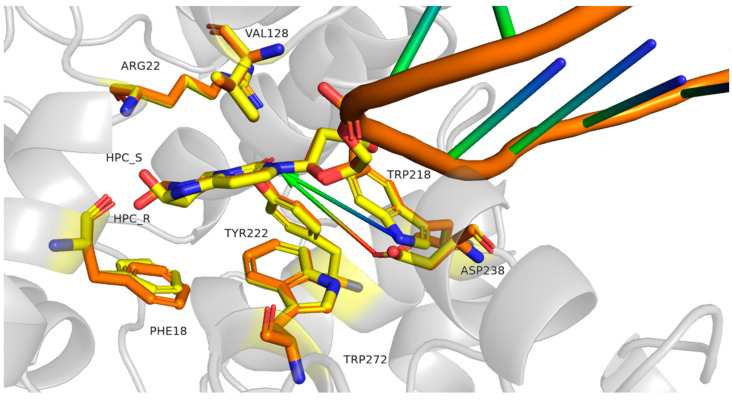
The active site of AlkA modeled in complex with DNA containing HPC. The residues proximal to the S- and R-HPC stereoisomers are depicted in yellow and orange, respectively, and arrows indicate the contribution of Trp218 and Asp238 in glycosidic bond cleavage.

Modification of cytosine by ACR results in the formation of two stereoisomers (R and S), owing to the chirality of the carbon atom bearing the hydroxyl group (Figure 1). Based on the available crystallographic structures of the double-stranded DNA (dsDNA) bound by AlkA [29,56], we modeled the hypothetical position of both HPC stereoisomers within the active site of AlkA in complex with dsDNA, as depicted in Figure 5.

**Figure 6:**
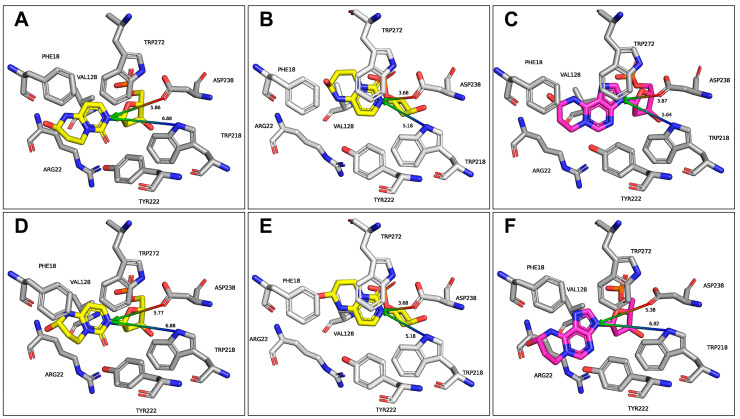
Comparison of the modeled complexes of AlkA with HPC(S) (**A**,**B**) and HPC(R) (**D**,**E**) with previously reported [21] in silico models of AlkA complexes with HPA(S) (**C**), and HPA-R (**F**). Panels (**B**,**C**,**E**) support an S_N_1-like glycosylase mechanism, while (**A**,**D**,**F**) support an S_N_2-like glycosylase mechanism, in which an additional water molecule contributes to glycosidic bond hydrolysis. HPC and HPA are denoted in yellow and magenta, respectively.

Both isomers adopt an anti-conformation and fit well into the active site pocket. The binding energies were estimated with FoldX (ver. 5) [57] to 12.8 and 13.4 kcal/mol for R and S stereoisomers, respectively. No steric hindrances have been identified. Numerous AlkA residues are involved in the ligand recognition (Phe18, Arg22, Gly123, Ser127, Val128, Trp218, Tyr222, Asp238, Tyr239, Leu240, and Trp272), with some of them involved only for one type of isomer—e.g., Ala21 and Tyr273 interact with isomer S, and Leu19 with isomer R. For both HPC isomers, the glycosidic bond is positioned close to the catalytic residues Trp218 and Asp238, adopting an arrangement that promotes acid-catalyzed cleavage of this bond. In this configuration, the enzyme is unlikely to distinguish between the R and S HPC stereoisomers. The placement of the modified bases within the active site is consistent with an S_N_2-like glycosylase mechanism, where a water molecule contributes to the excision of the base (Figure 6A,D), however alternative poses, in which the key residue Asp238 directly facilitates the cleavage of the glycosidic bond (i.e., S_N_1-like mechanism), are also possible (Figure 6B,E). The binding modes of the two forms are highly similar, with a minute preference for the S form (0.6 kcal/mol according to FoldX), as the hydroxyl group of the R form causes a slight displacement of Phe18. However, no such displacement is observed for the S_N_1-like conformation.

Comparison of the obtained structures with those previously determined for HPA (Figure 6) [21] shows that the orientation of the glycosidic bond is highly similar across the structures. The Tyr22 and Trp272 residues are closer to the modified pyrimidine base, suggesting a more rigid positioning within the active site. Nevertheless, the overall binding pattern remains conserved.

The binding poses of HPC and HPA differ substantially from the canonical conformation observed in B-DNA. Thus, despite the sugar moiety adopts the conformation characteristic for the B-DNA and the glycosidic bond in an anti-conformation with χ equaling 218° and 210° for R and S stereoisomers of HPC, respectively, the overall conformation of the backbone: *gauche*^+^ (α)-*trans* (β)-*gauche^+^* (γ)–C2′-endo (δ)-*gauche*^−^ (ε)-*trans* (ζ) (all dihedrals defined according to the IUPAC-IUB definition [58]) deviates from that commonly observed in B-DNA: *gauche*^−^-*trans*-*gauche^+^*-C2′-endo-*trans-gauche*^−^. Moreover, both HPC χ conformations differ slightly from those previously reported for the new AlkA substrate, HPA [21], which are 180° and 164° for the S and R stereoisomers, respectively. However, the χ values derived from in silico models of HPC bound at AlkA catalytic site agree with conformations observed in B-DNA (200 ÷ 240°), and with a forcefield parameterization commonly used in DNA refinement (230 ± 18° and 237 ± 24° for pyrimidines and purines, respectively [59]), so the HPC accommodation to AlkA does not introduce significant steric hindrances.

According to the QM calculations of Foloppe et al. [60], there is no conformational penalty for placing the HPC pirymidine ring at the binding site of AlkA (the optimal χ is 207° for the sugar moiety in S *gauche*^+^ conformation). In contrast to the latter, the conformations of both HPA stereoisomers (180 and 164°) differ from the optimal value of 230°, a deformation that may destabilize the glycosidic bond, thereby increasing susceptibility to AlkA-related hydrolysis. Moreover, the glycosidic bond is, generally, more stable in pyrimidine nucleosides than in purine ones [61]. It was also reported that AlkA exhibits low affinity for non-alkylated substrates and shows a stronger preference for cleaving electron-deficient purines, whose weakened glycosidic bonds are readily hydrolyzed under acidic conditions. Nevertheless, even alkylated substrates that are not electron-deficient (those lacking destabilization of the glycosidic bond) were not excised from the DNA structure [29].

In summary, both structural and electrochemical properties indicate that the glycosidic bond in HPC is substantially less susceptible to hydrolysis than that in HPA, despite the same type of modification in the same chemical context, including both geometry and topology.

## 3. Materials and Methods

### 3.1. Bacterial Strains

*E. coli* strains wt, and *alkA*, *alkB*, and double *alkAalkB* mutants have already been described and used [26].

### 3.2. Plasmid Modification

In vitro ACR (Fluka, Buchs, Switzerland) modification of pIF102, pIF103, and pIF104 plasmids was performed as described elsewhere [20].

### 3.3. Mutagenicity Assay

Preparation of electrocompetent cells, induction of the adaptive response, and electrotransformation were performed as described in [26]. Briefly, ACR-modified or mock-treated plasmids were introduced by electroporation into cells prepared in two versions: with an induced (by pretreatment with 1 mM methyl methanesulphonate (MMS, Aldrich St. Louis, MO, USA) or uninduced Ada response. The dilutions of the transformation mixture were spread onto LB plates supplemented with chloramphenicol (30 μg/mL) to estimate the total number of transformed cells and onto lactose minimal plates to select *Lac^+^* revertants. Transformation efficiency was defined as the number of colony-forming units (*cfu*) produced by 1 μg of plasmid DNA in a transformation reaction. Mutation frequency (MF) indicates the number of revertants per 10^4^ transformed cells.

### 3.4. Statistics

Statistical analysis was performed as described in [21]. We used the Statistica package (StatSoft Inc., Tulsa, OK, USA, 2011, STATISTICA, version 10, www.statsoft.com). Since the Shapiro–Wilk test indicated non-Gaussian distributions of the variables, the Kruskal–Wallis test, and pairwise differences in mutagenesis were analyzed with a post hoc Mann–Whitney U test (Figure 2). Differences with *p* < 0.05 after appropriate Bonferroni correction were considered statistically significant.

### 3.5. Purification of AlkA Glycosylase

AlkA was purified as described in [21].

### 3.6. Preparation of HPC-Containing Oligodeoxynucleotide

The reaction mixture contained 30 nmole of 5′-d(TTT TTT CTT TTT CTT TTT CTT TTT T)-3′ (T_22_C_3_) oligodeoxynucleotide (Metabion, Martinsried, Germany), 0.65 M sodium acetate, pH 4.5, and 1.5 M ACR in a final volume of 100 μL. The mixture was incubated for 15 min at 37 °C. Only cytosine residues could be modified in those conditions, as thymine is unreactive in an acidic solution [52]. The oligodeoxynucleotide was purified from excess ACR by precipitation with 70% ethanol, lyophilized, and redissolved in TE buffer.

### 3.7. Annealing

Eight nmole of unmodified (T_22_C_3_, control) or modified [T_22_(HPC)_3_] oligodeoxynucleotide was associated with 16 nmole of the complementary one 5-d(AAA AAA GAA AAA GAA AAA GAA AAA)-3′ (A_22_G_3_) in 0.1 M NaCl in the final volume of 50 μL. The mixture, preheated in a 90 °C water bath, was slowly cooled down to room temperature.

### 3.8. Enzymatic Excision of HPC

Excision of HPC by AlkA and enzymatic hydrolysis of double-stranded oligodeoxynucleotides were carried out according to [21].

### 3.9. Obtaining the HPC Standard

HPC was prepared by reacting deoxycytidine with ACR: a mixture of 15 µL of ACR (15 M, 95% aq. solution) and 0.6 mg of deoxycytidine was incubated in 400 µL of 0.6 M acetate buffer (pH 4.5) at 37 °C for 1 h. The product purity was confirmed by HPLC (Knauer, Berlin, Germany) as described previously [19], using a 0–100% linear gradient of 20 mM ammonium formate (Fluka, Buchs, Switzerland), pH 6.5, with 20% aqueous acetonitrile (J.T. Baker, Radnor, PA, USA) over 30 min.

### 3.10. HPLC Analysis

HPLC was performed as described in [21], but detection was carried out at the respective maximum absorbance wavelengths: 268 nm for dC and dT; 257 nm for dA; 250 nm for dG; and 281 nm for dHPC.

### 3.11. Molecular Modeling of AlkA in Complex with dsDNA Containing T(HPC)T

The initial geometries of both 3,N^4^-α-hydroxypropanocytosine stereoisomers were adopted from cytosine, with the coordinates of the exocyclic hydroxypropane adducts initially determined using the MP3 method and subsequently refined at the B3LYP/6-31G(d,p) level with the Firefly program [62]. The optimized geometries, together with ESP-derived atomic charges for both HPC stereoisomers, were incorporated into the Yasara2 force field for use in molecular modeling. Such an approach has already been successfully applied to analogous heterocyclic systems [19,21,63,64]. An atomic-resolution model of dsDNA containing T(HPC)T in complex with AlkA glycosylase was adopted from the T(HPA)T-containing DNA duplex bound to AlkA [21]. The geometry of 3,N^4^-α-hydroxypropanocytosine was prepared as described above. Molecular modeling of protein-DNA complexes was performed using the Simulated Annealing protocol in the YASARA-Structure package (YASARA Biosciences GmbH, Vienna, Austria), employing the modified Yasara2 force field [65]. Processing and visualization of crystallographic structures were performed using the PyMOL Molecular Graphics System (version 2.5.8), Schrödinger, LLC (New York, NY, USA).

## 4. Conclusions and Perspectives

Acrolein forms several types of DNA adducts, including the well-studied hydroxypropanoguanines and the less characterized lesions 1,N^6^-α-hydroxypropanoadenine (HPA) and 3,N^4^-α-hydroxypropanocytosine (HPC). Our previous studies showed that HPA is effectively repaired in vivo, in vitro, and in computer models by both AlkB dioxygenase and AlkA glycosylase [20,21].

In this study, we provide evidence that the acrolein adduct to cytosine, previously shown in vitro and in silico to be repaired by AlkB [19], is a mutagenic, non-instructive lesion. We also confirmed that it is repaired in vivo by AlkB, even without prior activation of the Ada response. Mutation patterns indicate that HPC primarily causes C→A transversions, followed by C→T transitions and C→G transversions. Despite its structural similarity to HPA, HPC is not a substrate for AlkA glycosylase. To understand this difference, we used molecular modeling to compare the accommodation of the two acrolein adducts within the AlkA substrate-binding pocket. Both R and S isomers of HPC adopt an anti-conformation and fit well into the active site; however, the glycosidic bond in HPC is much more stable than in HPA, making AlkA-mediated hydrolysis of the glycosidic bond unsusceptible.

While the chemical and biological properties of acrolein adducts to guanine are relatively well understood [3,10,11,12,44], HPA and HPC remain understudied. Previous research has shown that HPC is formed in both single- and double-stranded DNA after exposure to acrolein [14], but it likely occurs more frequently in ssDNA since the N3 and N^4^ positions of cytosine are protected in dsDNA by Watson–Crick pairing. It has also been demonstrated that HPC can be removed from dsDNA by *E. coli* Mug (less efficiently than εC) and, to some extent, by fission yeast Thp1p, but not by human TDG glycosylase [49]. However, there is no data on its repair mechanisms in higher organisms, on how the lesion is recognized, or on the structural effects of HPC on DNA, and many related issues. Since acrolein is produced endogenously as a byproduct of oxidative stress, HPC, like other related lesions, could influence processes linked to inflammation, chronic infections, and cancer. Therefore, continued investigation of human DNA repair enzymes, including their substrate specificities and mechanisms of action, is warranted. Insights derived from simple model organisms such as *E. coli* remain indispensable for elucidating how these lesions are recognized and processed in more complex biological systems.

## Data Availability

The raw data supporting the conclusions of this article will be made available by the authors on request.

## Supplementary Materials

The following supporting information can be downloaded at: https://www.mdpi.com/article/10.3390/ijms27010071/s1.

## Abbreviations

The following abbreviations are used in this manuscript:

Ada	adaptive response
ACR	acrolein
CAA	chloroacetaldehyde
ε	etheno
HPA	1,N^6^-α-hydroxypropanoadenine
HPC	3,N^4^-α-hydroxypropanocytosine
MMS	methyl methanesulfonate
m	methyl
MF	mutation frequency
SMF	Spontaneous mutation frequency
wt	wild-type
