# Supplementary material for: Mutagenicity and Repair of Acrolein Adduct to Cytosine
Source: Int J Mol Sci. 2025 Dec 21;27(1):71. doi: 10.3390/ijms27010071 (PMC12785776; doi:10.3390/ijms27010071)
Supplement: Supplementary file 1 [file ijms-27-00071-s001.zip › ijms-4012641-supplementary.pdf]

Table S1. Frequencies of ACR-induced mutations in the *lacZ* gene of **pIF102** plasmid indicatory for GC→AT substitutions replicated in various *E. coli* strains.

Strain	ACR concentration used for plasmid modification (mM)				
	0	5	10	25	50
	Frequencies of ACR-induced mutations ($\times 10^{-4}$)[a]				
wt	2.4 ± 1.1	3.5 ± 1.5	8.1 ± 2.1	25.5 ± 12.9	49.1 ± 25.4
wt$_{ad}$[b]	2.2 ± 1.1	3.3 ± 1.5	5.3 ± 1.3	15.6 ± 6.0	17.8 ± 11.1***
alkA	3.1 ± 1.6	3.9 ± 1.7	10.0 ± 3.6	28.4 ± 10.4	48.1 ± 10.7
alkA$_{ad}$	3.7 ± 0.9†††	3.3 ± 1.4	4.8 ± 2.3***	17.8 ± 5.9**	22.7 ± 9.8***
alkB	2.7 ± 1.8	11.6 ± 4.0†††	28.0 ± 8.1†††	114.6 ± 38.7†††	181.7 ± 99.5†††
alkB$_{ad}$	1.8 ± 1.7	8.8 ± 3.6†††	25.7 ± 6.1†††	36.2 ± 14.9***,††	100.9 ± 49.9†††
alkAalkB	2.3 ± 1.4	14.9 ± 7.2 †††,+++	40.6 ± 2.0 †††	164.7 ± 77.7 †††	nd[e]
alkAalkB$_{ad}$	3.5 ± 0.8	19.5 ± 5.3 †††,+++,###	142.5 ± 21.1 ***,†††,+++,###	288.8 ± 65.3 **,†††,+++,###	

[a]Data are expressed as mean ± SD.

[b]The "ad" subscript denotes strain adapted by MMS pretreatment.

[c]Statistical significance of difference between non-adapted and adapted strain is denoted by asterisks (** P<0.01, *** P<0.001); whereas

†† (P<0.01), ††† (P<0.001) refers to comparison between each mutant strain and wt strain[d];

++ (P<0.01), +++ (P<0.001) refers to comparison between *alkA and* double *alkAalkB* mutant[d];

(P<0.01), ### (P<0.001) refers to comparison between *alkB and* double *alkAalkB* mutant[d].

[d] – strains were compared pairwise non-adapted *vs* non-adapted and adapted *vs* adapted.

[e] nd – not determined.

Table S2. Frequencies of ACR-induced mutations in the *lacZ* gene of **pIF103** plasmid indicatory for GC→CG substitutions replicated in various *E. coli* strains.

Strain	ACR concentration used for plasmid modification (mM)				
	0	5	10	25	50
	Frequencies of ACR-induced mutations ($\times 10^{-4}$)[a]				
wt	2.7 ± 1.2	9.8 ± 5.6	16.1 ± 7.5	22.1 ± 7.1	53.3 ± 30.0
wt$_{ad}$[b]	1.3 ± 0.9	5.5 ± 3.3**	6.2 ± 3.8***	11.5 ± 5.3***	17.9 ± 10.1***
alkA	4.8 ± 1.6†††	4.6 ± 2.6	20.5 ± 7.9	26.2 ± 4.7	41.5 ± 6.2
alkA$_{ad}$	3.0 ± 1.1***,†††	3.3 ± 1.4	9.5 ± 0.7	14.6 ± 6.6**	20.9 ± 9.4***
alkB	4.2 ± 1.5††	49.3 ± 35.1†††	59.0 ± 19.1†††	104.2 ± 51.6†††	221.7 ± 125.4†††
alkB$_{ad}$	2.1 ± 0.9***	17.9 ± 9.7 †††	50.5 ± 15.1 †††	92.1 ± 28.5†††	194.8 ± 42.4†††
alkAalkB	15.4 ± 12.1	72.6 ± 21.5 $^{†††,+++}$	93.8 ± 55.3 $^{†††,+++}$	138.9 ± 81.9 $^{†††,+++}$	nd[e]
alkAalkB$_{ad}$	25.6 ± 19.9	102.0 ± 33.0 $^{†††,+++,\#\#\#}$	131.4 ± 63.9 $^{†††,+++,\#\#\#}$	193.2 ± 86.6 $^{***,†††,+++,\#\#}$	

[a]Data are expressed as mean ± SD.

[b]The "ad" subscript denotes strain adapted by MMS pretreatment.

[c]Statistical significance of difference between non-adapted and adapted strain is denoted by asterisks (** P<0.01, *** P<0.001); whereas †† (P<0.01), ††† (P<0.001) refers to comparison between each mutant strain and wt strain[d];

$^{++}$ (P<0.01), $^{+++}$ (P<0.001) refers to comparison between *alkA and* double *alkAalkB* mutant[d];

$^{\#\#}$ (P<0.01), $^{\#\#\#}$ P<0.001) refers to comparison between *alkB and* double *alkAalkB* mutant[d].

[d] – strains were compared pairwise non-adapted *vs* non-adapted and adapted *vs* adapted. [e]nd – not determined.

[e] nd – not determined.

Table S3. Frequencies of ACR-induced mutations in the *lacZ* gene of **pIF104** plasmid indicatory for GC→TA substitutions replicated in various *E. coli* strains.

Strain	ACR concentration used for plasmid modification (mM)				
	0	5	10	25	50
	Frequencies of ACR-induced mutations (× 10^{-4})[a]				
wt	2.7 ± 0.8	3.2 ± 0.9	8.5 ± 2.9	15.5 ± 4.4	29.3 ± 14.2
wt$_{ad}$[b]	1.9 ± 1.3	2.5 ± 1.1	4.4 ± 2.2**	6.6 ± 2.1***	12.1 ± 5.7***
alkA	3.1 ± 0.9	4.8 ± 1.1	9.6 ± 4.9	51.1 ± 16.5†††	74.2 ± 21.2†††
alkA$_{ad}$	4.1 ± 0.6†††	2.0 ± 0.7***	8.1 ± 1.9††	28.1 ± 6.3***,†††	22.8 ± 7.3***,†††
alkB	3.4 ±3.1	29.0 ± 15.3†††	108.4 ± 23.2†††	132.1 ± 51.5†††	310.8 ± 110.1†††
alkB$_{ad}$	2.0 ± 1.4	19.6 ± 4.1†††	90.7 ± 44.9†††	121.7 ± 53.8†††	287.3 ± 78.5†††
alkAalkB	6.9 ± 2.1 $^{\dagger\dagger\dagger,+++}$	34.3 ± 22.9 $^{\dagger\dagger\dagger,+++}$	139.8 ± 67.1 $^{\dagger\dagger\dagger,+++}$	191.2 ± 38.2 $^{\dagger\dagger\dagger,+++,\ \#\#\#}$	nd[e]
alkAalkB$_{ad}$	6.4 ± 3.2 †††	61.9 ± 65.7 $^{\dagger\dagger\dagger,+++}$	222.5 ± 147.4 $^{\dagger\dagger\dagger,+++}$	146.1 ± 54.5 $^{\dagger\dagger\dagger,+++}$	

[a]Data are expressed as mean ± SD.

[b]The "ad" subscript denotes strain adapted by MMS pretreatment.

[c]Statistical significance of difference between non-adapted and adapted strain is denoted by asterisks (** P<0.01, *** P<0.001); whereas

†† (P<0.01), ††† (P<0.001) refers to comparison between each mutant strain and wt strain[d];

$^{++}$ (P<0.01), $^{+++}$ (P<0.001) refers to comparison between *alkA and* double *alkAalkB* mutant[d];

$^{\#\#}$ (P<0.01), $^{\#\#\#}$ (P<0.001) refers to comparison between *alkB and* double *alkAalkB* mutant[d].

[d] – strains were compared pairwise non-adapted *vs* non-adapted and adapted *vs* adapted.

[e]nd – not determined.
